# Intramedullary Nailing of Femoral Shaft Fractures: Implications of Interlocking Screw Breakage

**DOI:** 10.7759/cureus.115721

**Published:** 2026-09-03

**Authors:** Eric T Metzman, Josue Balbuena, Taylor T Utter, Rodolfo Zamora

**Affiliations:** 1 Orthopaedic Surgery, University of Louisville, Louisville, USA

**Keywords:** femur intramedullary nail, femur nonunion, femur shaft fracture, hardware failure, intramedullary nail, nonunion femur fracture

## Abstract

Introduction

Femoral shaft fractures are commonly treated with intramedullary nailing (IMN), though complications such as delayed union, nonunion, and hardware failure remain significant challenges. Interlocking screw breakage has unclear implications for fracture healing. Our study examined patients with femoral shaft fractures treated with IMN who developed interlocking screw breakage to evaluate outcomes and complications.

Methods

The retrospective cohort study included 551 patients with femoral shaft fractures (AO Foundation (AO)/Orthopaedic Trauma Association (OTA) 32A/B/C) treated with IMN at a level one trauma center. Inclusion criteria were acute fractures treated definitively with IMN and a minimum one-year follow-up. Patient demographics, fracture characteristics (including OTA classification), and radiographic union at one year were recorded. Radiographs were reviewed preoperatively, postoperatively, and at clinical follow-up visits.

Results

Interlocking screw failure occurred in 31 patients (5.6%), of whom 24 had complete one-year follow-up. A total of 185 patients were included in the final analysis (24 with and 161 without screw breakage). There were no significant differences in age (48.5 vs 46.0 years, *p* = 0.545), BMI (27.8 vs 28.8, *p* = 0.509), smoking (41.3% vs 40.5%, *p* = 0.927), or diabetes (*p* = 0.41). Delayed healing at one year was observed in 66.7% of patients with screw breakage versus 37.9% without (*p* = 0.013). Among those with screw failure, 36.7% (n=11) had OTA type C fractures, and 41.94% (n=13) required revision surgery for nonunion (n=7), symptomatic hardware (n=4), or infection (n=2).

Conclusion

Interlocking screw breakage was associated with delayed healing and an increased likelihood of reoperation. Further investigations should focus on risk factor identification for screw breakage for prevention and proper patient counseling.

## Introduction

Femoral shaft fractures remain a common yet challenging problem for the orthopedic surgeon. The total number of global femoral shaft fractures continues to rise, with an estimated increase of 35% between 1990 and 2021 [1]. These fractures traditionally fall into one of two patterns: low-energy mechanisms resulting in spiral or oblique fracture patterns, and high-energy mechanisms resulting in transverse, comminuted, and open fractures [2]. Treatment for femoral shaft fractures is commonly reamed, closed intramedullary nailing (IMN) with static locking screws. The construct has proven to be a successful treatment option, achieving a union rate ranging from 85-100% at one year [3,4], with a consensus nonunion rate of 3-5% [5].

Among known complications for IMN of femoral shaft fractures, the significance of distal interlocking screw breakage, termed “auto-dynamization”, remains elusive. To date, there is scarce data on its rate in femoral shaft fractures and its significance. Rates of auto-dynamization vary, with a range of 4-10% in previously published work, dating from the 1990s and early 2000s [6,7]. Previous work has identified multiple factors contributing to interlocking screw breakage: improper technique resulting in increased stress, positioning too close to the fracture, and insufficient screw length or diameter [6]. The rate of auto-dynamization has not been updated with advancements in orthopedic IMN technology. The significance of auto-dynamization is unknown and can pose significant challenges with subsequent surgeries [8].

The primary purpose of this study was to identify patients with femoral shaft fractures treated with IMN who developed interlocking screw breakage within one year of follow-up and determine the rate of screw breakage at one year. Secondary objectives were to compare patient demographics, comorbidities, fracture classification, surgical outcomes, and union rates of patients with interlocking screw breakage to those without it.

## Materials and methods

This was a retrospective cohort study from a database of isolated femoral shaft fractures (AO Foundation (AO)/Orthopaedic Trauma Association (OTA) 32A/B/C) treated definitively with IMN at a single level one trauma institution, University of Louisville Hospital, in Louisville, Kentucky, United States, between 2017 and 2024. All surgeries were performed by fellowship-trained orthopedic trauma surgeons. Surgeries were identified with Current Procedural Terminology (CPT) code “27506”.

Study population

There were 551 patients in the database. Our inclusion criteria for the final study analysis were a minimum of one year of clinical follow-up and definitive fixation with IMN. Exclusion criteria included plate fixation and less than one year of clinical follow-up if the primary outcome was not observed.

Data collection

Patient demographic data, including BMI, sex, age, and medical comorbidities, such as smoking and diabetes, were gathered for all eligible patients.

Patient radiographs were reviewed by a single reviewer at multiple time points: preoperative, immediately postoperative, and follow-up at two weeks, six weeks, three months, six months, and one year. The reviewer was blinded to all patient complications. Fractures were classified using the OTA classification system. The side (right, left), location (proximal, midshaft, distal) of the fracture, and direction of the fixation (antegrade, retrograde) were documented. Broken interlocking screws were identified, with location and postoperative date recorded. Radiographic fracture healing (soft callus, hard callus, healed) was documented at all follow-up visits (Figures 1-3).

**Figure 1:**
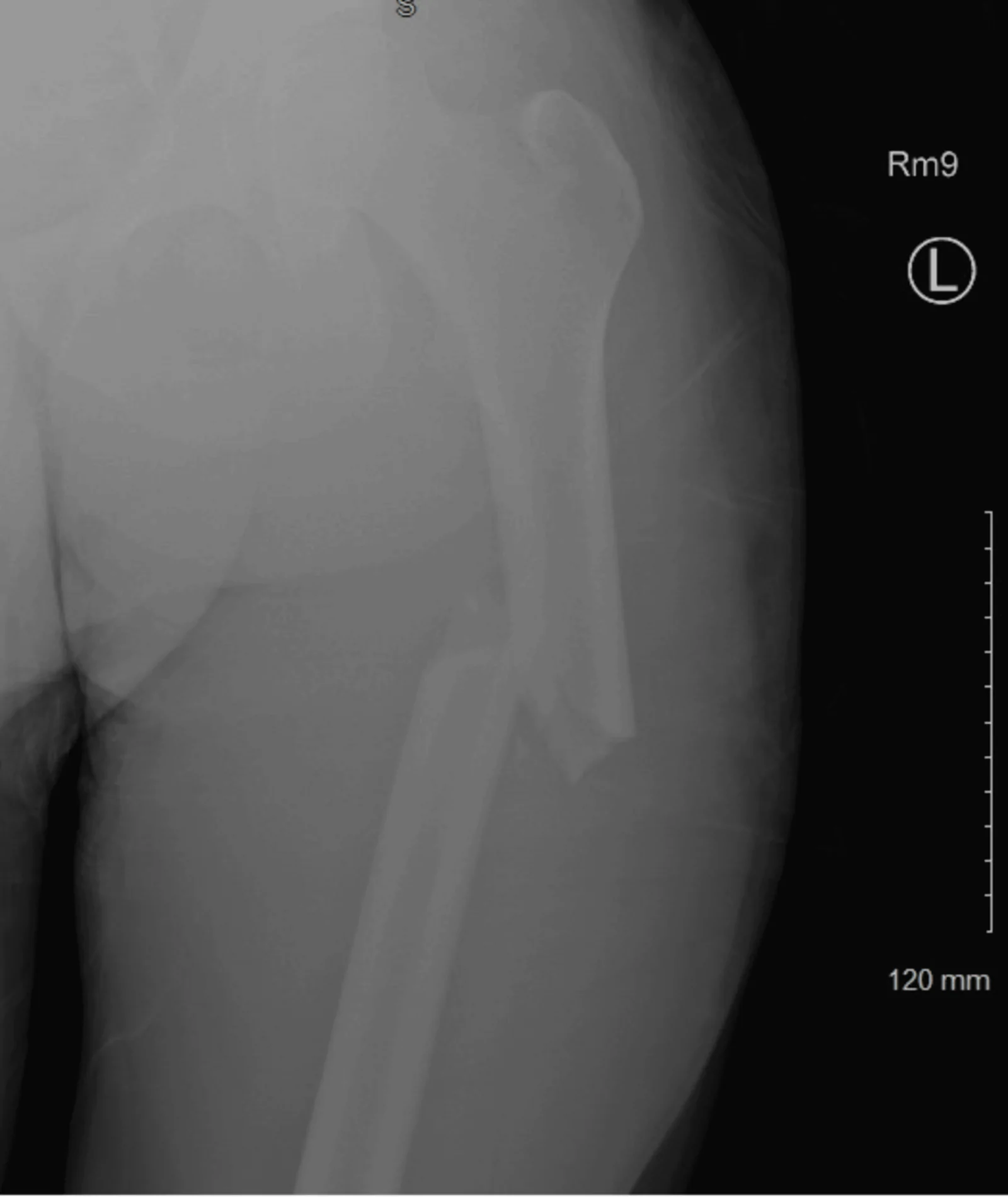
Radiograph (anteroposterior view) of a femoral shaft fracture subsequently treated with intramedullary nailing.

**Figure 2:**
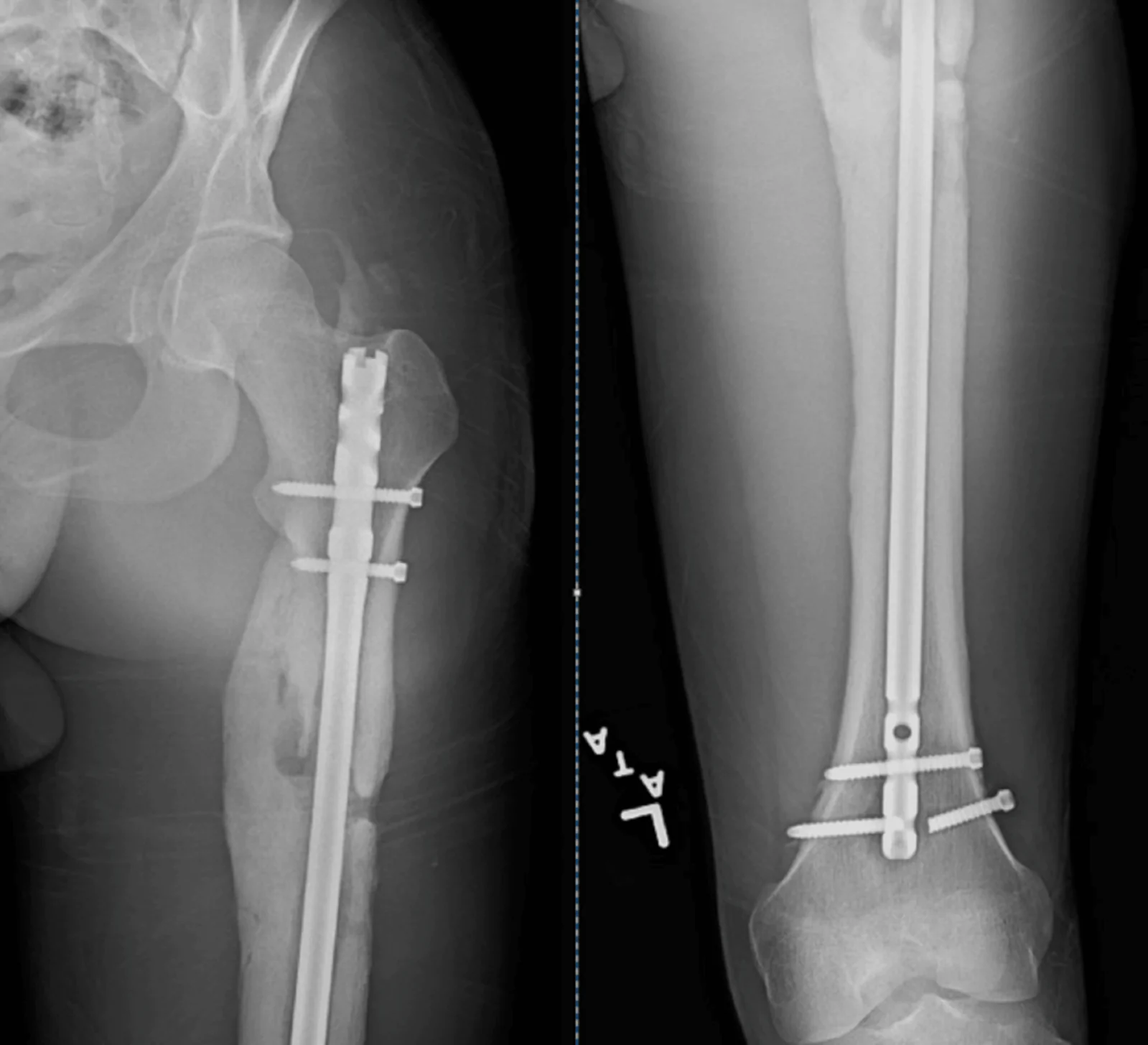
Two radiographs (anteroposterior view) of a femoral shaft fracture treated with intramedullary nailing at one year postoperative that underwent nonunion with broken interlocking screws

**Figure 3:**
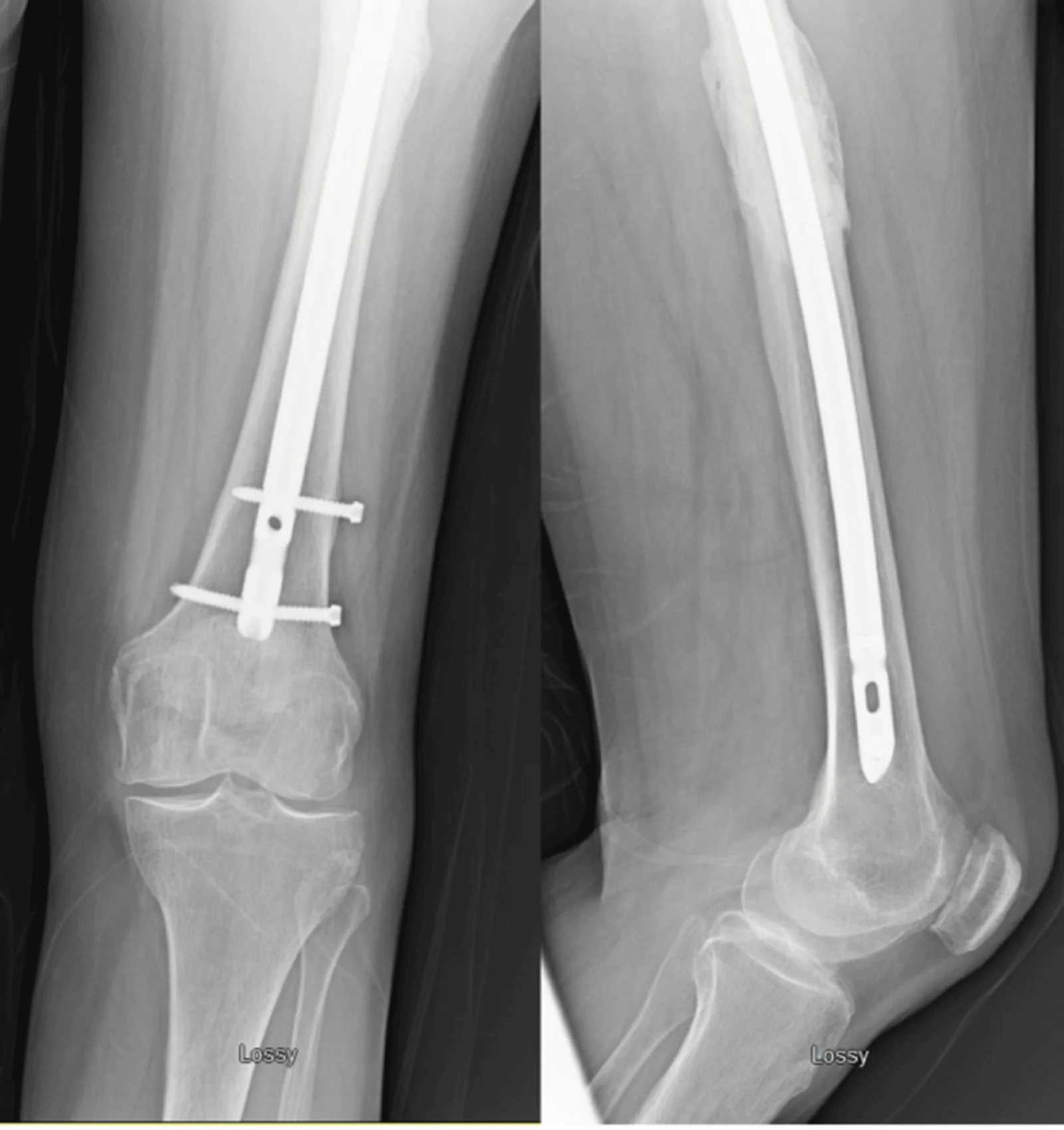
Radiographs (anteroposterior and lateral views) of a femoral shaft fracture treated with intramedullary nailing one year postoperative with union and a broken interlocking screw

Questions regarding healing classification were reviewed by a fellowship-trained orthopedic trauma surgeon, who evaluated the patient radiographs, and a decision was made after thorough discussion. All patient notes and operative records were reviewed to identify subsequent second surgeries, regardless of death or length of follow-up.

Definition

We defined a healed fracture as solid cortical bridging of at least three cortices between the fracture fragment(s) on anteroposterior (AP) and lateral X-rays.

Data analysis

Data collection and statistical analysis were performed using Microsoft Excel (Microsoft Corporation, Redmond, Washington, United States). Statistical tests included *t-*tests, Chi-squared tests, and Fisher's exact tests as indicated. We performed Fisher's exact tests rather than Chi-squared tests for calculations with two-by-two tables and expected counts less than or equal to 5.

## Results

For the 551 patients reviewed, the average age at injury was 46.15 years, with an average BMI of 28.78 kg/m^2^. A total of 213 reported smoking, and 71 reported diabetes. A total of 31 (5.63%) were identified to have broken interlocking screws (study group), and those without were placed in the control group. Between the study and control groups, there was no difference in age (48.52 vs 46.01, p = 0.545), BMI (27.84 vs 28.84, p = 0.505), smoking (41.3% vs 40.5%, p = 0.944), and diabetes (6.45% vs 13.3%, p = 0.41). A majority (n=20, 64.5%) of the patients with broken screws received antegrade intramedullary nails (Table 1).

**Table 1:** Comparison between broken and nonbroken screw groups (N=551) *P*-value significance set to <0.05. Fischer exact tests and *t*-tests were performed for statistical analysis *There were 24 patients with interlocking screw failure in the study group

Variable		Broken Screws (n=31)	Nonbroken Screws (n=520)	Test statistic value (if applicable)	P-value
Female sex, n (%)		10 (32.26%)	204 (39.23%)	-	p = 0.570
Age (years), mean ± SD		48.52 ± 21.33	46.01 ±22.50	t = 0.605	p = 0.545
BMI, mean ± SD		27.85 ±5.66	28.84 ±8.23	t = 0.667	p = 0.505
Smoker, n (%)	Yes	12 (38.71%)	201 (38.65%)	-	p = 0.927
	No	17 (54.83%)	295 (56.73%)		
	Unspecified	2 (6.45%)	24 (4.62%)		
Diabetes mellitus, n (%)	Yes	2 (6.45%)	69 (13.27%)	-	p = 0.41
	No	29 (93.55%)	449 (86.35%)		
	Unspecified	0 (0%)	2 (0.39%)		
Antegrade Nail, n (%)		20 (64.5%)	286 (55%)	-	P = 0.34
Radiographic union at 1 year (n=185), n (%)		8/24* (33.3%)	100/161 (62.1%)	-	p = 0.013

A total of 185 patients had a minimum one-year clinical follow-up; of these, 24 (14.9%) were from the study group (broken screws). Among the 24 patients from the study group who had a one-year follow-up, 16 (66.66%) were not healed at one year, compared to 61 of the 161 control group patients (37.89%), p = 0.013. Table 1 summarizes findings between the two groups.

OTA classifications are included in Table 2. The most common fracture pattern in both the study and control groups was A (n=14 (46.7%) and n=82 (58.2%), respectively), followed by C (n=11 (36.7%) and n=44 (31.2%), respectively) and B (n=5 (16.7%) and n=44 (10.6%), respectively). No statistical differences were noted between the two groups (p = 0.454).

**Table 2:** OTA classification between broken and nonbroken screw groups *P*-value significance set to < 0.05. Chi-squared test was performed for statistical analysis. OTA: Orthopaedic Trauma Association

OTA Classification	Broken Screws	Nonbroken Screws	Test Statistic Value (if applicable)	P value
A	14 (46.7%)	82 (58.2%)	χ^2 ^= 1.58	p = 0.454
B	5 (16.7%)	15 (10.6%)		
C	11 (36.7%)	44 (31.2%)		

Of the 24 study group patients (interlocking screw failure) who went through a one-year follow-up, eight were healed at the one-year follow-up. The study group patients without healing at one year had more complex fracture patterns compared to those with healing (OTA 32C; n=8 vs n=1, p = 0.174), though this did not reach statistical significance. All patients with broken screws who healed at one year had screw breakage distal to the fracture site (10 out of 10 broken screws), though this did not reach clinical significance when compared to the not-healed group (p = 0.014). Table 3 summarizes the findings between healed and not-healed study group patients with a minimum of one year of follow-up.

**Table 3:** Distribution according to healing status of study group patients who underwent the one-year follow-up radiographic evaluation follow-up (N=24) P-value significance set to < 0.05. Chi-squared test and Fisher's exact test were performed for statistical analysis. *Calculations done according to number of screws

Parameters		Healing Status at 1 Year		Test statistic value (if applicable)	P-value
		Healed (n = 8)	Callus Only (n = 16)		
Total number of broken screws		10	21		
Broken screw location compared to fracture*, n (%)	Proximal	0/10 (0%)	5/21 (23.81%)	-	p = 0.14
	Distal	10/10 (100%)	16/21 (76.19%)		
Nail type, n (%)	Antegrade	7/8 (87.50%)	10/16 (62.50%)	-	p = 0.35
	Retrograde	1/8 (12.50%)	6/16 (37.50%)		
Fracture location, n (%)	Proximal femur	0/8(0%)	1/16 (6.25%)	χ^2 ^= 1.09	p = 0.580
	Femoral shaft	8/8 (100%)	14/16 (87.50%)		
	Distal femur	0/8 (0%)	1/16 (6.25%)		
Fracture type, n (%)	Open	1/8 (12.50%)	3/16 (18.75%)	-	-
	Periprosthetic	1/8 (12.50%)	2/16 (12.50%)	-	-
OTA Classification, n (%)	A	6/8 (75%)	6/16 (37.50%)	χ^2 ^= 3.5	p = 0.174
	B	1/8 (12.50%)	2 /16 (12.50%)		
	C	1/8 (12.50%)	8/16 (50%)		

A total of 13 patients (41.94%) underwent a second operation, with etiologies being nonunion repair (n=7), symptomatic hardware (n=4), and infection (n=2). Two nonunion repairs required subsequent redo revision surgery.

## Discussion

Femoral shaft fractures are a common, challenging orthopedic case, complications of which can cause significant morbidity for the patient. This study sought to clarify a common complication of unknown significance: interlocking screw breakage. Our observed incidence (5.63%) was comparable to previously published studies that report an incidence of interlocking screw breakage of 4-10% [6,7].

Wu and Shih published a series of 274 femur fractures, including nine with a minimum follow-up of one year [6]. They found 13 patients with screw breakage (4.7% incidence). The authors postulated four factors promoting screw breakage: elevated stress concentration around the screw hole and nail slot, nicking of the nail during drilling of the screw holes, very close proximity of the screw hole to the fracture, and large loading over the proximal femur. They found that the incidence of screw breakage was higher in more proximal or more distal fractures, though the authors did not analyze union rate in the patient cohort. In contrast, Im et al. published a 9.7% incidence of interlocking screw breakage in a series of 72 patients [7]. They analyzed nail diameters and found a correlation between smaller nail diameters and increased screw breakage incidence. Though the sample size was small, the finding is in line with previous arguments presented by Wu and Shih [6] that increased stress increases the risk for screw breakage.

Our study observed a statistically lower union rate at one year in patients with broken screws compared to those with nonbroken screws (33.3% vs. 62.1%, p = 0.013). In conjunction with decreased union at one year, we observed a high complication rate associated with interlocking screw breakage; almost half (41.94%) of the study group required a second surgery for either infection, symptomatic hardware, or nonunion revision. Known risk factors for femoral shaft nonunion include AO/OTA classification, open fractures, postoperative weight-bearing status, current smoking, and nail direction [9]. Our study observed no differences in OTA classifications or smoking status between the two study groups, though, of note, 16.7% of the study group patients had an open fracture, thus increasing their risk for delayed union. Given the nature of our study, we could not conclude whether broken interlocking screws are related to the cause or the sequela of delayed union, though it was a poor prognostic indicator. Future work should attempt to identify risk factors related to interlocking screw breakage, such as fracture malreduction.

We distributed patients with broken screws according to healed status and found a noticeable trend of not-healed patients having more complex fracture patterns (OTA C, n=8 not healed vs n=1 healed). We hypothesize that the finding was restricted by the small sample size in each subgroup. In a series of 248 femoral shaft fractures treated with IMN by Metsemakers et al., there was an observed nonunion rate of 11.3% at one year [10]. They found that more complex fractures have higher risks of nonunion, with OTA grade C fractures having a higher incidence of nonunion compared to OTA grade A. Our study observed a higher delayed union rate (37.9%) in total and a comparably increased rate in patients with broken screws (66.6%). The findings of decreased union at one year and high reoperation rate highlight the increased risk of patients with broken interlocking screws. Our study indicates that these patients should be closely monitored and advised in the postoperative period.

Limitations

There are several limitations to the study. Our study was performed at one of the two adult level one trauma centers in the residing state. Many patients were transported to our institution for immediate care, then returned to their home community for postoperative follow-up. A significant portion of reviewed patients (n=366) had no postoperative clinical follow-up to review and were excluded from final analysis. It is possible our large loss-to-follow-up percentage was, in part, due to the patients having healed and recovered well from their initial surgery, prompting no return to the clinic. This is partially supported by the percentage of one-year follow-up in the broken screw group (24/31; 77.42%) compared to the entire study population (185/551, 33.58%). It would also explain a lower-than-expected union rate at one year in the nonbroken screw group (62.11%). Symptomatic patients seemed more likely to continue to seek care from the operative surgeon. If, as postulated, a majority of these patients were satisfied and asymptomatic, likely then without screw failure, the true incidence at one year would be lower than that observed within the study.

Other limitations include the retrospective nature of the study. Data were limited to that clearly contained within the electronic medical records. Confounding variables may have influenced the union rate observed, such as fracture complexity, smoking, nail type, nail technique, and fracture location. Though direct comparison was made between groups with regard to potential confounding variables, no multivariate analysis was performed.

## Conclusions

Our results suggest that broken interlocking screws are a poor prognostic indicator following femoral shaft fracture IMN and are associated with delayed fracture union and high risk for a second operation. We identified no obvious risk factors for interlocking screw breakage, though increased fracture complexity trended towards significance. Further work should focus on identifying risk factors for interlocking screw breakage to better prevent complications and counsel patients.
